# Effect of Temperature on the Rate of Ageing: An Experimental Study of the Blowfly *Calliphora stygia*

**DOI:** 10.1371/journal.pone.0073781

**Published:** 2013-09-03

**Authors:** Megan A. Kelly, Adam P. Zieba, William A. Buttemer, A. J. Hulbert

**Affiliations:** 1 School of Biological Sciences, University of Wollongong, Wollongong, Australia; 2 School of Health Sciences, University of Wollongong, Wollongong, Australia; 3 Centre for Integrative Ecology, Deakin University, Geelong, Australia; Uppsala University, Sweden

## Abstract

All organisms age, the rate of which can be measured by demographic analysis of mortality rates. The rate of ageing is thermally sensitive in ectothermic invertebrates and we examined the effects of temperature on both demographic rates of ageing and on cellular senescence in the blowfly, *Calliphora stygia.* The short lifespan of these flies is advantageous for demographic measurements while their large body size permits individual-based biochemical characterisation. Blowflies maintained at temperatures from 12°C to 34°C had a five to six-fold decrease in maximum and average longevity, respectively. Mortality rates were best described by a two-phase Gompertz relation, which revealed the first-phase of ageing to be much more temperature sensitive than the second stage. Flies held at low temperatures had both a slower first-phase rate of ageing and a delayed onset of second-phase ageing, which significantly extended their longevity compared with those at high temperatures. Blowflies that were transferred from 29°C to 15°C had higher first-phase mortality rates than those of flies held at constant 15°C, but their onset of second-phase ageing was deferred beyond that of flies held constantly at this temperature. The accumulation of fluorescent AGE pigment, a measure of cellular oxidative damage, increased steadily over time in all blowflies, irrespective of the temporal pattern of mortality. Pigment accumulated steadily during periods of ‘negligible senescence’, as measured by minimal rate of mortality, and the rate of accumulation was significantly affected by temperature. Thus accumulation of AGE pigment is more representative of chronological age than a reflection of biological age or a cause of mortality.

## Introduction

Ageing is a universal process that has been identified in organisms ranging from bacteria, to plants and animals [1]. Ageing is often described as a progressive decline in function and survival with time, but because something is correlated with age does not necessarily make it a causal mechanism. The methods best-suited for distinguishing the causes of ageing from age-related changes, are experimental manipulations that extend longevity through cellular and molecular processes conjectured to cause ageing. To date, the most common experimental treatment used to extend longevity has been caloric (or dietary) restriction, which over 80 years ago, when used as a treatment to influence the body size of rats, was discovered to give rise to increased longevity [2]. However, the earlier observation that low temperature extended the life span of *Drosophila*, an ectothermic animal [3], [4], preceded this first observation of caloric restriction extending longevity by almost two decades and, because of the well known effects of temperature on the metabolic rate of ectotherms, these studies were seminal to the development of the ‘rate of living theory of ageing’ [5]. The effects of low temperature are relatively little used in ageing research in recent times, most likely a result of this connection between temperature and the now disproved ‘rate of living theory’ [6].

While the most common animal models used to investigate ageing (rodents and the invertebrates; *Drosophila melanogaster* and *Caenorhabditis elegans*) have provided significant insights, because of its universality, it is important to investigate ageing in other species. Here we report on an investigation into the effects of temperature on ageing in adults of the blowfly *Calliphora stygia*. As has previously been suggested [7], [8], this insect species combines the experimental advantages of both the invertebrate models (short lifespan and cheap husbandry costs) and the rodent models (a body size sufficient for measurement of individuals).

Biological ageing (or senescence) is not simply time since birth, but is associated with the cumulative deterioration of physiological capacity to maintain homeostasis, which ultimately leads to death. The rate of ageing can therefore be measured as either the cumulative changes at a cellular level (cellular senescence), or the final product of senescence at the population level (demographic senescence). We have compared the effects of temperature on both types of senescence in this study.

Cellular senescence was examined by the measurement of advanced glycation end-product pigments (AGE pigments). This was because these auto-fluorescent compounds consist of a complex mixture of oxidatively damaged biomolecules, which accumulate irreversibly with age [9]. These compounds are characteristic of cell ageing [10] and are thought to contribute to the process of ageing by disrupting normal cell proteostasis [11]. It has been suggested that age-related pigments may be a source of oxidants in senescent cells, as well as likely having an effect on metabolic pathways of senescent cells [12], [13]. The accumulation of AGE pigments is also associated with a number of age-related diseases [9], [14], [15]. That they accumulate with age in a range of tissues, including muscle and neural tissues, and are found across species, makes these pigments highly useful measures of ageing [16]. Additionally, an examination of a range of damage-related biomarkers of ageing in *D. melanogaster* found AGE pigments to be the only biomarker that correlated with mortality rates [17].

Demographic senescence was examined using a two-phase Gompertz analysis [18], [19]. The Gompertz equation is the most common model used to describe demographic ageing, which expresses a linear relationship when the natural logarithm of mortality rate is plotted against age, and the slope of this relationship indicates the rate of ageing [20]. Some species, for example some turtles, deep sea fish and naked mole rats, show little or no increase in mortality rates with time and display periods of what has been termed ‘negligible’ senescence [21]. In our experiments, it became apparent that *C. stygia* held at low temperatures showed 'negligible' demographic senescence early in their adult life, followed by a faster increase in mortality later in life. We have therefore used a two-phase Gompertz equation to distinguish these two different phases of 'early' and 'late' ageing. The same demographic analysis has previously been used to differentiate early and late rates of ageing in species ranging from humans to flies [18], [19].

We used two approaches to examine the effect of temperature on ageing. The first was to compare ageing rates in populations of blowflies kept at different constant environmental temperatures, ranging from 12°C to 34°C, throughout their entire adult lives. The second approach was to examine the influence of a change in environmental temperature on ageing during adult life on populations of blowflies using a temperature-crossover experiment. Replicate populations of adult blowflies were swapped between 15°C and 29°C environmental temperature at both 14 days and 28 days post-eclosion. The responses of each of these populations were compared with the populations of blowflies maintained continuously at either 15°C or 29°C for their entire adult lives. This temperature-crossover approach has been used previously in experiments on ageing in *D. melanogaster* [17], [22].

## Methods

### General Methods

#### The study animal

Pupae of the eastern golden-haired blowfly (*Calliphora stygia*) were purchased from a commercial supplier (Sheldon’s Bait, Parawa, SA, Australia). Equal numbers of pupae were added to replicate cages prior to eclosion and flies were allowed to hatch over a two-day period, following which uneclosed pupae were removed. This meant that each fly present in the cage was of the same age (± one day), and we have therefore assigned date of eclosion for all flies to be the middle of this 48-hour period in our calculations. Overall population size (∼350 per cage) was not known until all flies in that cage had died.

Fly cages had aluminium frames (350 mm×400 mm×350 mm) fitted with a solid base and insect-screen on all but one side, which was covered with pantyhose (with the feet cut off) allowing hand access for changing food and water and removal of dead flies while preventing the escape of living flies. Flies were provided water *ad libitum*, and were given continual access to a standard sugar-yeast food mixture set in Petri dishes which was refreshed daily [8]. Each cage was provided with food always available in excess.

#### The food provided

The sugar-yeast diet is based on a *Drosophila* standard diet, which was previously shown to successfully maintain adult *C. stygia* [8]. It consisted of 200 g of sugar and 200 g of yeast (Enoferm M1 general wine yeast, Laffort Oenologie, Lallemand, Underdale, SA, Australia), 7.2 g of agar (Technical No.3, Oxoid, Adelaide, SA, Australia) and 0.5 g of nipagin to inhibit any bacterial or fungal growth (Sigma-Aldrich, Sydney, NSW, Australia). This mixture was dissolved in 400 ml of water and dispersed in 15 ml portions into Petri dishes.

### Main Experiment: Group cages

Two replicate cages were placed in six separate temperature- and humidity-controlled rooms. The regulated temperatures ranged from 12°C to 34°C and all were maintained at 60% relative humidity. Temperature and humidity were recorded continuously using Gemini Tinyview Dataloggers (Chichester, UK). An additional four cages were placed in two of the rooms (15°C and 29°C) as part of a temperature-crossover experiment, where two of the cages were swapped between these rooms at either 14 days or 28 days post-eclosion.

#### Measurement of food consumption

To estimate average daily food consumption per fly in each cage, food plates were weighed (±0.1 mg) before being placed in the cage and upon removal the next day using a Mettler Toledo AB204-5 balance. All food consumption values were corrected for evaporative water loss based on mass losses in control food Petri dishes placed under the same temperature and humidity conditions. Daily food consumption per fly was calculated by measuring the food mass per cage at the beginning and end of each day, and after correction due to mass loss by water evaporation, the total mass loss of food per day was divided by the number of flies in that cage on that day.

#### Measurement of egg laying

Eggs were often laid on the food dishes within the cages and the number present each day was recorded to gauge the timing and extent of female oviposition. This was used to determine the effect of each temperature treatment on normal function of female blowflies.

#### Measurement of longevity

Dead flies were removed and recorded daily along with their gender. These mortalities were used to create life tables using GraphPad Prism 5 (GraphPad Software, Inc., La Jolla, CA, USA). Differences in survivorship curves between treatments were examined for statistical significance using the Log-Rank (Mantel-Cox) test. For all treatments, survival rates in replicate cages were statistically indistinguishable, so data from replicate cages were pooled. Average longevity was calculated as the mean longevity of the population and maximum longevity calculated as the mean longevity of the top five percent of the longest-lived flies for each treatment, based on censuses of the pooled replicate cages.

#### Measurement of demographic senescence

To measure demographic senescence (the demographic rate of ageing), we used the Gompertz analysis of mortality curves derived from the life tables of pooled replicate cages. The Gompertz model, which describes an exponential increase in the rate of ageing with time, is expressed as µ_x_ = A.exp^Bx^, where A is the intercept and B is the slope or the change in mortality rate with time (i.e. the rate of ageing). Age-specific mortality (µ_x_) was calculated as: µ_x_ = 1-(l_x+1_/l_x_), and l_x_ is the fraction of animals surviving at day x. Mortality data was plotted as ln(µ_x_) and analysed using linear regression techniques. When the mortality data at lower temperatures were examined with this method, it was obvious that a single-phase Gompertz was inadequate to describe the mortality curve. There was a significant period of stable mortality before the onset of an exponential increase in daily mortality and, in order to quantify this early period of low senescence, we used a segmental linear regression technique (GraphPad Prism 5 Software, Inc, La Jolla, CA, USA) to fit a two-phase Gompertz curve as described by Carey [20].

The two-phase Gompertz showed two distinct phases, which we refer to as the ‘1^st^’ and ‘2^nd^’ phases of ageing and is described as µ_x_ = A_1._exp^B^1^x^ if×≤ bp/A_2._exp^B^2^(x–bp)^ if x> bp, where bp represents the day of the break point in the lines and describes the length of the period of early phase of ageing (B_1_). As with a regular Gompertz, the slope of the line is the rate of demographic senescence (B) as described by ln(µ_x_) = A+Bx for each phase of ageing. To gauge the significance of the segmental linear regression relationship for each temperature, an AICc comparison was used to compare the model with a simple Gompertz linear regression.

#### Measurement of cellular senescence

To evaluate cellular senescence, we measured a commonly used marker of ageing, the accumulation of fluorescent AGE pigment. We collected live flies throughout the experiment to determine their AGE pigment concentration and, where population numbers were sufficient, six flies were collected from each treatment at five time points. Flies maintained at high temperatures had shortened life spans and so were not collected at all time points. Flies removed from the populations while alive were not included in the calculation of life tables and survival curves. Fluorescent AGE pigment concentration was measured in both whole flies and, for another group, AGE pigment content of head, thorax and abdomen were evaluated and then summed to determine whole fly content. To examine the relationship between flies of varying ‘physiological health’ and AGE pigment content, a small sample of flies were also measured at 16 and 35 days. These flies were determined to be either ‘healthy’ (‘alive’ and flying about cage), ‘unhealthy’ (‘dying’ and lying on their backs and unable to right themselves) or ‘dead’.

Fluorescent AGE pigment determination was assayed using the methods of Oudes et al. [14] and Jacobson et al. [17]. Briefly, individual flies (or fly parts) were placed in a 1.5 ml Eppendorf tube, containing 900 µl of phosphate-buffered saline (PBS) with 10 mM Na_2_ ethylenediaminetetraacetic acid (EDTA) and homogenized using a hand-held homogeniser. Following homogenisation, 100 µl of trypsin solution (0.1 g of trypsin dissolved in 100 µl of PBS containing 10 mM EDTA) was added to the homogenate and tubes were then incubated for 24 hours at 37°C. The digested homogenate was centrifuged at 11000 *g* for five minutes, the supernatant removed and spin-filtered through a 0.22 µm cellulose acetate membrane (Costar spin-X, Corning, NY, USA) at 11000 *g* for five minutes. The filtrate was added to 96-well plates in 50 µl aliquots, which were made up to 200 µl by addition of 150 µl of PBS containing 10 mM EDTA. Fluorescence was measured using a FLUOstar Optima (BMG Labtech, Germany) at excitation and emission wavelengths of 360–10 nm and 440–10 nm, respectively. Samples were diluted to ensure they were within the linear range of fluorescence (as determined by a preliminary experiment). Each fluorescence measurement was corrected for background fluorescence and performed in triplicate. Individual AGE pigment concentration was taken as the mean fluorescence from triplicate wells, and where triplicates varied by more than five percent, averages were calculated from the two closest values. Inter-assay variation was determined using a standard sample and averaged 1.4%. Data measured on whole flies are expressed relative to fluorescence values measured on whole flies collected immediately post-eclosion, while data measured on fly body segments are expressed in fluorescence units.

### Supporting Experiment: Individual Housing

Flies were housed individually to determine their food consumption and longevity. In this experiment, nine males and ten females were placed into individual specimen jars (100 ml) maintained at 25°C and 60% relative humidity and plugged with foam stoppers to permit gas exchange [7]. These were provided with water and standard sugar-yeast food in separate small containers (1.5 ml Eppendorf tube caps). Food containers were replaced daily and weighed to determine food consumption. Five control containers devoid of flies were used to measure, and correct for, mass loss of the food due to evaporation of water.

### Statistics

To account for the potential increases in Type-I errors due to the large number of statistical comparisons made between temperature treatments, statistical significance was conservatively set to *P*<0.01 for ANOVA comparisons of the effects of constant temperature treatments on adult longevity, egg laying, food consumption, and cellular senescence. Statistical significance was kept at *P*<0.05 for analysis of individually kept flies and in analyses comparing within temperature treatments. Both single and two-phase Gompertz relations were fit to the demographic data of each temperature treatment, and the model most likely to generate the data was determined by Akaike’s Information Criterion (AIC). All datasets were analysed using GraphPad Prism 5 (GraphPad Software Inc., California, USA), with the exception of MANOVA comparisons, which were performed in JMP Pro 9 (SAS Institute Inc., North Carolina, USA).

## Results

### Effect of Temperature on Average and Maximum Longevity

Ambient temperature had a significant effect on survivorship in adult *C. stygia* (χ^2^ *= *2413, *df = *5, *P*<0.0001). Both average and maximum longevity were significantly extended when flies were maintained at lower temperatures (Table 1). Average longevity increased six-fold, from 15 days at 34°C to 91 days at 12°C, while maximum longevity was extended almost five-fold over this temperature range (longevity differences between genders are shown in Fig. S1).

**Table 1:** Longevity of adult *Calliphora stygia* maintained at different ambient temperatures.

Temperature (°C)	Average longevity (days)	Maximum longevity (days)	*N*
12°C	90.8±1.6^a^	155.8±1.5^a^	685
15°C	87.9±1.5^aA^	152.2±1.4^aA^	733
20°C	45.1±1.2^b^	105.6±0.7^b^	742
25°C	27.7±0.7^c^	66.9±0.7^bc^	747
29°C	23.2±0.5^cC^	55.4±1.5^cdE^	757
34°C	15.2±0.2^d^	32.0±0.6^d^	777
Transfer from 29°C to 15°C			
At 14 days	47.0±1.5^B^	143.1±1.5^AB^	782
At 28 days	25.6±0.8^C^	99.6±2.8^BC^	790
Transfer from 15°C to 29°C			
At 14 days	32.2±0.5^B^	61.8±1.2^DE^	709
At 28 days	45.4±0.5^D^	71.0±0.8^CD^	785

All values are means ± SEM. Maximum longevity is calculated as the average longevity of the five % longest-lived animals for each group. Average and maximum longevities are compared between temperatures by Kruskal-Wallis ANOVA with temperature pairs compared using Dunn’s multiple comparison post-hoc test (Average longevity: *K-W = *1984, *df = *5, *P*<0.0001; Maximum longevity: *K-W = *200.8, *df = *5, *P*<0.0001). Flies undergoing temperature transfer were compared to the 15°C and 29°C populations by Kruskal-Wallis ANOVA with temperature pairs compared using Dunn’s multiple comparison post-hoc test (Average longevity: *K-W* = 1984, *df = *5, *P*<0.0001, Maximum longevity: *K-W* = 200.0, *df = *5, *P*<0.0001). Values that do not share the same letter are statistically significantly different (*P*<0.01), lowercase letters are used for comparisons between the constant temperature populations, uppercase letters are used for comparisons between the temperature transfer experiments.

### Effect of Temperature on Food Consumption

**Figure 1:**
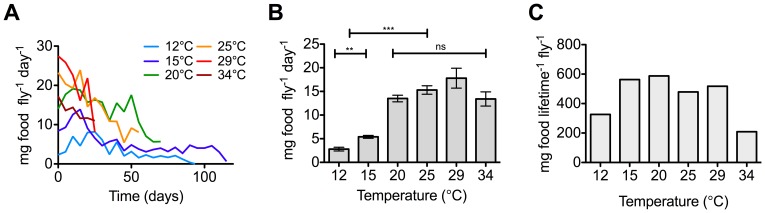
Food consumption of adult *Calliphora stygia* maintained at different constant temperatures. (A) Food consumption over time of flies maintained at the six different ambient temperatures. Data are from pooled replicate cages and averaged over a five-day period. Error bars are omitted for clarity. (B) Average daily food consumption of flies was significantly less at very low temperatures (12°C and 15°C), however there were no significant differences in average food consumption between the moderate to high temperatures (20°C to 34°C). Values are means ± SEM. ** is *P*<0.001; *** is *P*<0.0001. (C) Average lifetime food consumption per fly was calculated as the average daily food consumption per fly multiplied by average lifespan (in days).

Total food consumption was measured for each population cage and then divided by the number of flies present to calculate the average daily food consumption per individual blowfly (Fig. 1A). There was a similar pattern in daily food consumption over the lifespan of the six different populations, with daily food consumption being higher initially and then decreasing over time. An ANOVA (food consumption as a dependent variable, days as a covariate and temperature as factor, *F*_11, 388_ = 54.1, *P*<0.0001) showed a significant effect of time (*F*_1,1_ = 25.3, *P*<0.0001) and temperature (*F*_5, 5_ *= *24.8, *P*<0.0001) on food consumption, as well as a significant interaction between time and temperature (*F*_5, 5_ = 8.7, *P*<0.0001). Because food consumption rate was measured at a population level and not an individual fly level, there are two alternate explanations for this decrease in food consumption with time. Firstly, the most obvious explanation is that individual flies reduce their food consumption with age. Alternatively, if there is a large variation between individual flies in food consumption and flies consuming large amounts of food have a shorter lifespan than those consuming low amounts of food, a consequence of this will be that over time the calculated average food consumption per fly decreases. This may be called a ‘rate of living’ effect, assuming that food consumption per individual is a correlate of its metabolic rate (i.e. its ‘rate of living’). Of course, the explanation could also be a combination of these two. In order to differentiate between these two explanations, the food consumption of individually housed flies was measured over time at 25°C.

**A test of the ‘rate of living’ effect: food consumption and longevity of blowflies individually housed at 25°C.**

**Figure 2:**
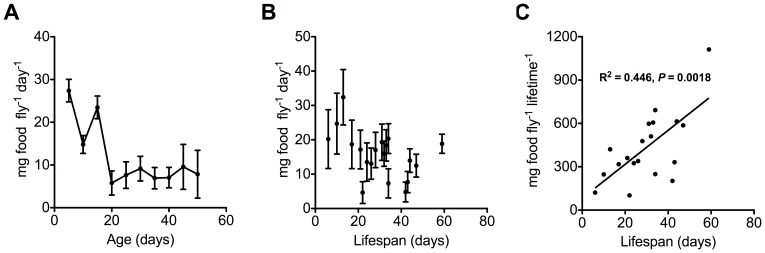
Relationship between food consumption and lifespan of individually-maintained flies. Daily food consumption of flies that were kept individually at 25°C (*N = *10 females and 9 males). (A) Average daily food consumption of adult flies fed *ad libitum*. Data points are averaged over all flies for a 5-day period, error bars are ± one SEM. (B) Average food consumption of each individual over their entire lifetime showed no negative relationship between average daily food consumption and lifespan as would be predicted by the rate of living theory (*F*_1, 17_ = 4.115, *P*>0.05). Data points are the average daily food consumption of an individually-maintained fly, with error bars ± one SEM. (C) Total lifetime food consumption was calculated per individual by multiplying average food consumption by total longevity. There was a significant positive relationship between lifetime food consumption and lifespan (*F*_1, 17_ = 13.7, *P*<0.01).

The daily food consumption and longevity of individual blowflies maintained at 25°C is shown in Figure 2. These results support our first interpretation, in that flies quite uniformly decreased food consumption as they got older. Individually housed flies showed a similar decrease in daily food consumption with time as when food consumption was measured at the population level at 25°C (Fig. 2A compared to Fig. 1A). An ANOVA of individually measured food consumption showed significant effects of time (*F*_1, 554_ *= *36.3, *P*<0.001). Furthermore, there was little variation in food consumption between individuals (see error bars in Fig. 2A), which further dismisses the second explanation.

The ‘rate of living’ theory predicts a ‘live-fast die-young’ relationship. If a high metabolic rate necessitates high food consumption, it predicts a negative relationship between food consumption and individual longevity. However, average daily food consumption was not inversely related to individual longevity when examined collectively (*F*_1,17_ = 4.115, *P>*0.05; Fig. 2B). Similarly, the rate of living theory also predicts constant lifetime energy consumption among flies, that is, flies have the same lifetime food consumption irrespective of longevity. However, contrary to this prediction of no relationship, there was a strong positive relationship between total lifetime food consumption and total lifespan for individuals (*F*_1,17_ = 13.70, *P*<0.01; Fig. 2C).

Measurements of individual food consumption at 25°C were statistically indistinguishable from estimates of food consumption rates at the population level (also held at 25°C; Welch-corrected *t* = 0.62, *df = *241, *P*>0.05). Individually housed flies had an overall average daily food consumption of 14.5±1.0 mg per fly, and flies in the population cages held at 25°C consumed an average of 15.3±0.9 mg per fly.

### Effect of Temperature on Average and Lifetime Food Consumption

For each temperature, daily food consumption was averaged over the period of measurement (Fig. 1B) and this average daily food consumption was combined with the average longevity (Table 1) to provide a calculated ‘average lifetime food consumption’ per individual fly for each of the six temperatures (Fig. 1C). Average daily food consumption was significantly influenced by temperature (Kruskal-Wallis test = 202.4, *df = *5, *P*<0.0001), with food consumption rate significantly lower at 12°C and 15°C compared to all other temperatures (Dunns multiple comparison post-hoc test *P*<0.0001, Fig. 1B). Food consumption rate was highest at 29°C, with 17.9±2.1 mg food consumed per fly per day, and lowest at 12°C, with only 2.8±0.4 mg food consumed per fly per day. While there was a general increase in average daily food consumption with increasing temperature, there was no significant difference in food consumption rates between populations held at temperatures at or above 20°C (Dunn’s multiple comparison post-hoc test *P*>0.05).

Average lifetime food consumption also showed relatively little difference between flies maintained at 15°C and 29°C (Fig. 1C). There was a large decrease in average lifetime food consumption in flies held at the two extremes of 34°C and 12°C (204–254 mg food per fly per lifetime), being almost half that of the more moderate temperatures (413–608 mg food per fly per lifetime).

### Effect of Temperature on Egg Laying

**Figure 3:**
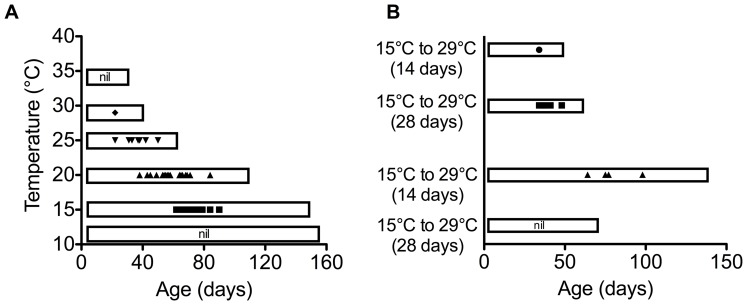
Frequency and duration of egg laying of female *Calliphora stygia* kept at different ambient temperatures. Individual data points represent the days on which the presence of eggs on food dishes was recorded. The box around the data indicates the female maximum lifespan for each temperature treatment. (A) Flies kept at constant ambient temperatures (*n = *351; 388; 379; 402; 399; and 401 from 12°C to 34°C respectively). (B) Flies undergoing temperature transfer regime. The red dotted line shows the time of the temperature transfer (*n = *389, 397, 399, 399, 376, and 388, listed from the top to bottom categories respectively).

Egg laying was used as an indicator that conditions of housing and food allowed normal physiological function. There were no eggs layed at either the highest temperature of 34°C or the lowest temperature of 12°C (Fig. 3A). This indicates that the temperature range we employed spanned the entire physiological range of *C. stygia.*

The age at first egg laying decreased with increasing temperature, with flies at 25°C beginning egg laying at 22 days post-eclosion, while flies maintained at 15°C began 40 days later (Fig. 3A). However, when expressed as a percent of female maximum longevity, the reproductive period was similar for all temperatures, with the median egg-laying day occurring at 49%, 56%, 64% and 54% of the maximum longevity at 15°C, 20°C, 25°C and 29°C, respectively. When scaled to maximum longevity, the length of time over which females were reproductive was shortened at 15°C and 29°C, with only one laying day observed at 29°C. Females at 15°C laid for only 19% of their maximum longevity, while in comparison females kept at 20°C and 25°C were reproductive for up to 50% of their maximum longevity.

### Effect of Temperature on Demographic Senescence

**Figure 4:**
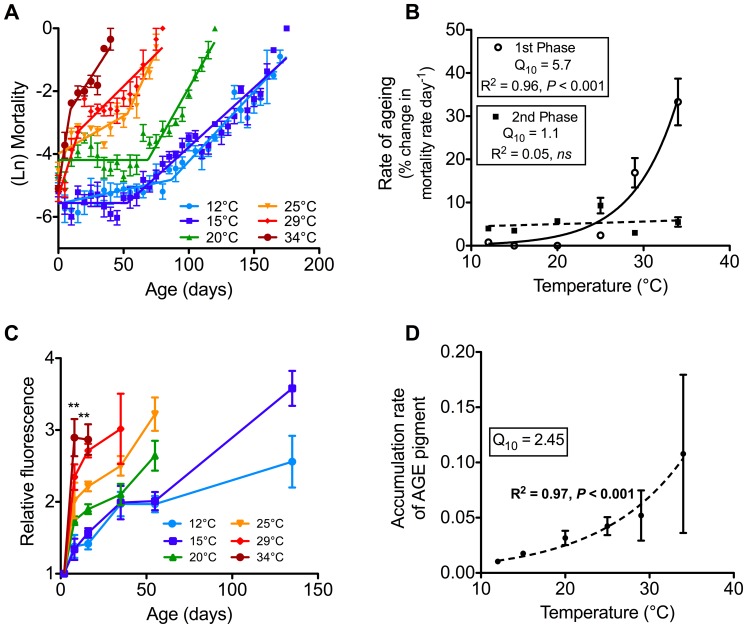
Demographic and cellular senescence of adult *Calliphora stygia* maintained at different ambient temperatures. (A) A two-phase Gompertz was fitted to daily log-transformed mortality by segmental regression and is represented by the solid line for each temperature (see Table 2 for parameters). Data are pooled from replicate cages, with plotted points being the average of a five-day period, with error bars ± one SEM (*N* = 12°C = 685, 15°C = 733, 20°C = 742, 25°C = 746, 29°C = 618, and 34°C = 778). (B) The exponential relationship between 1^st^ and 2^nd^-phase rate of ageing and temperature. Data points are the average (± one SEM) rate of ageing during 1st- and 2^nd^-phases of ageing. (C) Fluorescent AGE pigment accumulation with chronological age for each temperature treatment. Values are means ± SEM (*N* = 6). ** represents a significant difference between temperatures at that age. (D) Temperature sensitivity of AGE pigment as described by the *Q*_10_ of the rate of accumulation of fluorescent AGE pigment. Data points are the means ± SEM of the rate of accumulation at each temperature.

While initial mortality rates of populations were not significantly different between temperature treatments (Table 2), the changes in age-specific mortality with time were significantly affected by temperature (Fig. 4A; gender-specific mortality rates are shown in Fig. S1). All mortality curves were better fit by a two-phase Gompertz than with a single-phase Gompertz (as determined by AIC comparisons; Table 2). For temperatures ≤20°C, the rate of ageing (i.e. the slope of the line) during the 1^st^ phase was not significantly different from zero, indicating negligible demographic senescence occurring during this phase at low temperatures. While there was no statistically significant increase in mortality rates during the 1^st^ phase at these low temperatures, there was a significant difference in the level of mortality. Populations kept at 20°C had a higher level of mortality during this early period than those kept at 12°C and 15°C.

**Table 2:** Mortality parameter estimates from a two-phase Gompertz fit to daily mortality of adult *Calliphora stygia* maintained at different ambient temperatures.

Temperature(°C)	Initial mortality%(% mortality per day)	Duration of 1^st^phase of ageing(days)	Rate of 1^st^-phaseageing (% change inmortality rate per day)	Rate of 2^nd^-phaseageing (% change inmortality rate per day)	Single vs.two-phaseGompertz (R^2^)
12	0.6±0.4^a^	83±6^a^	0.8±0.3^a#^	4.0±0.3^a^	0.72 vs. **0.81**
15	0.6±0.1^a^	48±5^b^	−0.6±0.6^a#^	3.5±0.2^b^	0.78 vs. **0.84**
20	0.9±0.7^a^	61±4^ab^	−0.4±0.5^a#^	5.7±0.6^c^	0.49 vs. **0.68**
25	1.1±0.2^a^	55±4^b^	2.4±0.4^b^	9.3±1.8^d^	0.66 vs. **0.74**
29	0.6±0.3^a^	17±3^c^	16.9±3.4^c^	3.0±0.6^e^	0.64 vs. **0.77**
34	0.6±0.1^a^	10±1^c^	33.3±5.4^d^	5.5±1.1^abc^	0.72 vs. **0.88**
Transfer from 29°C to 15°C					
14 days	0.7±0.3	83±4^C^	0.0±0.0^#B^	4.6±0.4^B^	0.50 vs. **0.71**
28 days	0.9±0.2	72±8^AC^	1.5±0.4^#C^	6.0±1.4^C^	0.54 vs. **0.63**
Transfer from 15°C to 29°C					
14 days	0.8±0.4	na	na	5.15±0.5^BC^	0.64 vs. na
28 days	0.3±0.3	21±5^B^	10.8±2.0^D^	4.81±0.9^BC^	0.79 vs. **0.82**

% Initial mortality, calculated as the average mortality over the first five-days, was invariant between constant temperatures as compared by Kruskal- Wallis ANOVA (*K-W = *0.65, *df = *5, *P*>0.05).

Daily mortality data were fitted by both single linear regression and segmental linear regression, the latter giving two phases of ageing (1^st^ and 2^nd^) and a time point at which the two phases intersect (length of 1^st^ period). For each temperature treatment these two equations were compared by an AICc comparison to determine the most likely model to have generated the data, R^2^ values are given for each fit. In the temperature transfer experiment, mortality curves were applied immediately following the transfer.

For each mortality parameter, populations are compared between temperature pairs by an *F-*test (*P*<0.01). Values that share the same letter are not significantly different (lowercase letters compare constant temperatures, uppercase letters are used to compare between temperature transfer populations).

# The 1^st^ phase of ageing was also tested to determine whether the rate of ageing (i.e. the slope of the line) was significantly different from zero. Slopes that were not significantly different from zero are denoted by ^#^.

For temperatures ≥25°C, there was significant demographic senescence during the 1^st^ phase, as the slope was significantly greater than zero, and this rate increased at higher temperatures. As well as the rate of early ageing being temperature dependent, the duration of this phase was also influenced by temperature (Table 2). Furthermore, the duration of the 1^st^ phase decreased with higher temperature (Fig. 4A).

**Figure 5:**
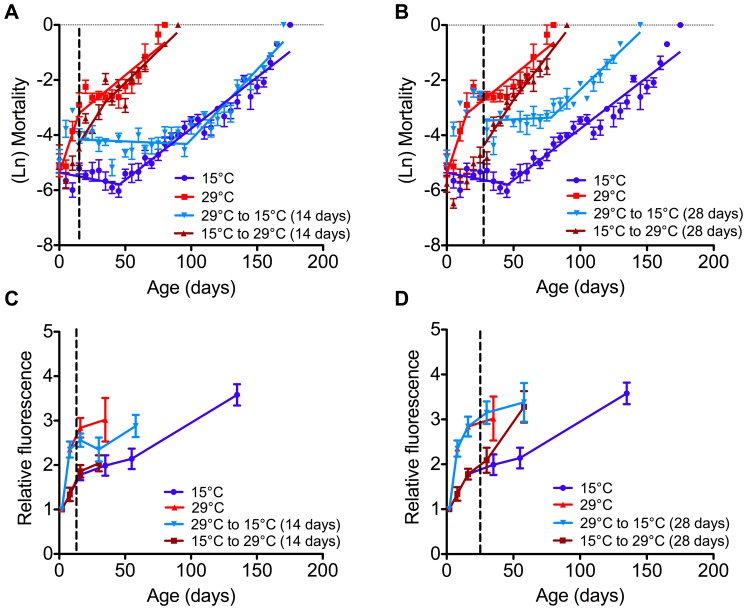
Cellular and demographic senescence of adult *Calliphora stygia* transferred between high (29°C) and low (15°C) temperatures. (A) Age-specific mortality of flies that were transferred between temperatures after 14 days. Data are presented with data points calculated as an average of a five-day period (error bars are omitted for clarity). See Table 2 for analyses of slopes. (B) Age-specific mortality of flies that were transferred between temperatures after 28 days. Data are presented with data points calculated as an average of a five-day period (error bars are omitted for clarity). See Table 2 for analyses of slopes. (C) Fluorescent AGE pigment accumulation in whole body samples of flies transferred between temperatures after 14 days (represented by the vertical dotted line). Data points are means ± one SEM (*N* = 6). (D) Fluorescent AGE pigment accumulation in whole body samples of flies transferred between temperatures after 28 days (represented by the vertical dotted line). Data points are means ± one SEM (*N* = 6).

The 2^nd^ phase of demographic senescence showed much less temperature sensitivity than the 1^st^ phase (Fig. 5 and Table 2). For example, in the 1^st^ phase, the change in mortality rate varied from zero percent to 33% per day, while during the 2^nd^ phase, the change in mortality rate varied from three to nine percent per day. At temperatures at or below 25°C, the rate of ageing during the 2^nd^ phase was faster than that during the 1^st^ phase, however at temperatures above 25°C, the rate of ageing was slower during the 2^nd^ than during the early phase of ageing.

The relationship between temperature and the rate of a physiological process can be expressed as a *Q*_10_. Examining the relationship between temperature and the rate of ageing (Fig. 4B), shows there was a strong effect of temperature on the 1^st^ phase of ageing, but almost no temperature effect on the 2^nd^ phase. The 1^st^ phase of ageing had a *Q*_10_ of 5.7, which is very high compared to typical biological processes (such as metabolism or food consumption), which generally have *Q*_10_’s between 2 and 3 [23]. In contrast, the 2^nd^ phase of ageing had a *Q*_10_ of 1.1, which indicates almost no temperature sensitivity.

### Effect of Temperature on Cellular Senescence

Cellular senescence was measured as the accumulation of fluorescent AGE pigment in whole body or body segments. Initially, AGE pigment was measured in homogenised whole flies and the change in fluorescence expressed relative to the level found in newly emerged adults (Fig. 4C). We later measured AGE pigment accumulation separately in head, thorax, and abdomen and expressed these in fluorescence units (Fig. S2). In all cases, newly emerged flies had measurable levels of AGE pigment present that were presumably accumulated during larval and pupal life. After eclosion, there was a rapid increase in AGE pigment at all temperatures early in adult life (*F*_6, 35_ *= *16.3, *P*<0.0001). Fluorescent AGE pigment accumulated throughout periods of ‘negligible demographic senescence’ that were observed during the 1^st^ phase of ageing at low temperatures.

Temperature directly affected the rate of fluorescent AGE pigment accumulation (Fig. 4C). When the rate of pigment accumulation in whole flies is averaged over the total experimental period, the relationship between temperature and accumulation rate displayed a *Q*_10_ of 2.5 (Fig. 4D). Measurement of the change in AGE pigment content of the body segments showed this measure of cellular senescence to accumulate predominantly in the head but not the thorax nor abdomen of individual flies. Approximately 90% of AGE pigment was in the head, with little measured in the abdomen (∼8%) and levels in the thorax being close to the level that could be reliably measured (see Fig. S2). The rate of accumulation of AGE pigment in heads was faster at higher temperatures, with a *Q*_10_ of 2.6 matching the thermal sensitivity of whole body AGE pigment accumulation.

Accumulation of AGE pigment in flies of the same chronological age may however, include flies of varying physiological health. If AGE pigment accumulation is affected by an individual’s health, this may result in differences in AGE pigment levels that mask age-related correlations. To address this possibility, we measured AGE pigment in three groups of flies that were defined as: alive, dead and almost dead (lying on their back and unable to turn over), at two different times (16 days and 35 days), in flies held at 25°C (see Fig. S3). These results were somewhat inconclusive, as while there was a significant difference between alive and dead flies at day 16 (P<0.01), there was no difference by day 35 (P>0.05). Similarly, there was also no difference between dying and dead flies at either day 16 or day 35 (P>0.05).

### Temperature-crossover Experiment

In all cases of temperature transfer, average and maximum longevity were altered to a value intermediate to the longevity of the two constant temperature groups (15°C and 29°C), with the degree of change in longevity dependent on age at the time of transfer (Table 1).

The effect of temperature transfer on mortality rates is presented in Figure 5, where two-phase Gompertz equations were fit to daily mortality rates following the day of transfer. Temperature had a very rapid effect on mortality rate at both low and high temperature, with changes to mortality patterns evident immediately following temperature transfer. The transfer of flies to the low temperature at both 14 and 28 days resulted in a period of ‘suspended’ demographic senescence, which was at a level of mortality unchanged from the previous high mortality experienced prior to transfer from high temperature (Figs. 5A and 5B).

Fly age at the time of transfer had an effect on the 2^nd^ phase of ageing, with flies transferred to low temperature at 28 days having a faster rate of ageing than those transferred at 14 days and those transferred at 14 days having a faster rate of ageing than those maintained constantly at 15°C (Table 2). The age when transferred also affected the ageing rates of flies transferred from low to high temperature (15°C to 29°C). While both populations experienced a rapid increase in mortality rates with exposure to the high temperature, the flies transferred at 14 days showed no slowing of the rate of ageing with old-age, while flies transferred after 28 days and those held constantly at 29°C did exhibit a slower rate of 2^nd^-phase ageing (Table 2).

Overall, the rate of ageing during the 2^nd^ phase was similar for all transferred flies (five to six percent change per day in mortality rate), but large differences in maximum longevity (up to a two-fold increase) due to the duration of ‘negligible’ senescence experienced by flies transferred to low temperature. Flies that experienced a cross-over all showed a significantly faster rate of 2^nd^-phase ageing compared to the flies that did not experience a temperature cross-over, which had a 3- 3.5% change per day in mortality rate (Table 2).

Egg laying was affected by both temperature and age at transfer (Fig. 3B). For the flies transferred at 14 days, their subsequent egg laying resembled that of the flies kept constantly at their transfer temperature. Flies transferred to 15°C layed eggs for the same amount of time as those kept constantly at 15°C, although they had fewer laying days. Flies transferred to 29°C had only one egg laying event. Flies transferred to 15°C after 28 days did not lay eggs. This may be a result of the period of egg laying already passing before the flies were transferred to 15°C. In contrast, flies that were transferred to high temperature at 28 days had increased frequency and duration of egg laying than those maintained constantly at 29°C and also laid eggs earlier and for a shortened period of time compared to flies kept constantly at 15°C.

Temperature transfer had limited effects on the accumulation of fluorescent AGE pigments (Figs. 5C and 5D). The most obvious change in pigment accumulation occurred in flies transferred to high temperature after 28 days, with a 50% increase in AGE pigment compared to those kept chronically at 15°C. There was no corresponding increase in accumulation in AGE pigment in flies transferred at 14 days. Flies transferred to low temperature after 14 days showed a small decrease in AGE pigment, yet this was not evident in the flies transferred after 28 days.

## Discussion

Temperature affected both demographic and cellular senescence of adult *C. stygia.* Demographic ageing for all temperature treatments was best described by a two-phase Gompertz equation, which has been used extensively in mortality studies in *Drosophila* [18], [24], [25], [26]. Across all temperatures, the 1^st^ phase of ageing was far more temperature sensitive than the 2^nd^ phase, but the strongest influence of temperature was the delayed onset of ‘normal’ demographic senescence observed at low temperatures, which resulted in an extended period of ‘negligible’ demographic senescence. A delay in the onset of senescence at low temperatures has previously been inferred from survival curves in other species including *Drosophila* [27], [28], however to our knowledge our study is the first one to quantify this phenomenon.

An increase in mortality rate with time is generally considered to be indicative of senescence. Surprisingly, we found temperature to have little influence on the rate of ageing of *C. stygia* during this period of their adult life. Thus while temperature had a dramatic effect on the 1^st^-phase rate of ageing and delayed the onset of ‘normal’ senescence, once senescence began, ageing occurred at a similar rate at all temperatures. Changes in thermal sensitivity over an insect’s adult life have been reported previously [29], [30], [31], [32], [33], and size (and thus age) has also been shown to influence the thermal tolerance of marine invertebrates [34].

The process of ageing (i.e. senescence) can be measured both demographically and at a cellular level. When determined demographically, mortality is the parameter measured. However, when cellular senescence is examined, the parameter measured may increase with age but this increase is not necessarily associated with a decline in function leading to death [35]. Accumulation of endogenous fluorescent compounds with age has been demonstrated in a range of vertebrates and invertebrates [36]. At all temperatures measured, there was an initial period of rapid increase in fluorescent AGE pigment, which was faster at high temperatures. Sheldahl and Tappel [37] also found faster accumulation of lipofuscin with increasing temperature in *Drosophila* flies, with a more rapid accumulation early in adult life. In *C. stygia*, this rapid initial increase in accumulation occurred even during the delayed ‘negligible’ demographic senescence that occurred at low temperatures. While fluorescent AGE pigment was the only marker of irreversible cellular damage that correlated with mortality rates in *D. melanogaster*, this was not measured at very low temperatures [17]. This lack of correlation between cellular senescence and demographic senescence suggests that although AGE pigment content may increase with age through chronological accumulation, it does not contribute directly to mortality [38].

There is a potential problem with determining the accumulation of AGE pigment in a live population, in that samples may include unknown proportions of physiologically young and physiologically old flies (i.e. close to death), and these may differ in their AGE pigment levels. Our attempt to address this problem by measuring AGE pigment in flies that were alive, dead and almost dead (lying on their back and unable to turn over), gave somewhat inconclusive results. While there was a significant difference between alive and dead flies at day 16, there was no difference by day 35, similarly, there was also no difference between dying and dead flies at either day 16 or day 35. Gerstbrein et al. [39] performed a similar examination of fluorescent AGE pigment in *C. elegans* that were age-matched but differed in physiological health. They found animals that they deemed to be less physiologically healthy and therefore closer to death had higher levels of pigment accumulation. They found that AGE pigment was predominantly found in the gut of *C. elegans*, which is unlike the situation we observed in the blowfly.

Accumulation of AGE pigment in *C. stygia* was confined to the head region, which has also been reported for the flesh-fly, *Sarcophaga bullata* [40] and house-fly, *Musca domestica* [41]. *Calliphora stygia* showed no accumulation of AGE pigment with age in the abdominal segment or thorax similar to results for flesh-fly abdominal tissue [40]. Ettershank et al. [34] also measured lipofuscin content of the larval phase and found the accumulation of lipofuscin with age in the larvae similar to that in the adult, with a loss of lipofuscin between the larval and pupal phases and again between the pupal and adult phases, suggesting some elimination of this pigment during transition between life history stages.

The separate measurement of fluorescent AGE pigment in different body segments allows consideration of how accumulation varies among different tissue types. This is because the thorax consists of mainly muscle, the abdomen contains primarily digestive and reproductive tissues, and the head comprises predominantly neural tissue. Mitochondrial DNA damage was found to be highest in the thorax of *Drosophila* [27], and muscles appear to be highly sensitive to ageing [42]. Yet, changes in physiological age, both chronologically and through an exposure to heat stress, were best described by changes in lipid profiles in the head of medflies, *Ceratitis capitata*, as compared to the other body segments [43]. Examination of fatty acid composition of the phospholipids of the head, thorax and abdomen of bees, showed that the peroxidation susceptibility of the heads of worker bees was double that of the other two segments [44]. These differences between tissue types in their response to ageing and their susceptibility to various ageing-related stressors highlight the complexity of the ageing process [45].

The variable effect of temperature during adult life has important implications for thermal adaptation during changing climate regimes [34]. In the face of climate change, there is a need for greater understanding of the interactions between temperature increase and biological processes [46]. It is well established that body temperature has a significant effect on the metabolic rates of ectotherms [47] which is expected to influence longevity in these organisms. Indeed, temperature effects on ectothermic longevity as according to the metabolic theory of ecology [48] have been suggested to explain much of the intraspecific latitudinal variation in lifespan [49].

However it has been repeatedly shown that there is not a simple inverse relationship between metabolic rate and longevity. For example, *Drosophila* with varying longevities show no relationship between their metabolic rate and lifespan [50], [51], [52], [53]. Similarly, at an individual level there is no evidence of a relationship between an individual’s lifespan and its metabolic rate, both in *Drosophila* [51] and in mice [54]. Dietary restriction was also initially thought to operate by reducing metabolic rate, however this has been shown not to be the case. Dietary restriction does not reduce mass-specific metabolic rate in mice [55], [56], *Drosophila* [7] or *C. elegans* [57]. It has also been shown in both humans and rats that voluntary exercise and its concomitant increase in metabolic rate does not decrease lifespan [58],[59].

The fact that egg laying occurred at all but the two extreme temperatures suggests we examined *C. stygia* over their complete physiological temperature range. In the absence of oxygen consumption measures, food consumption rate can be used as a proxy for metabolic rate under the conditional assumption that food consumption is related to metabolic needs. If food consumption does not match metabolic rate then there will be changes in body mass. A previous study using this species measuring food consumption found individuals to maintain body mass throughout their lifespan and showed similar changes in food consumption rates with age [7], and we have assumed this is also the case in the current study. Our measures of food consumption rates showed little effect of temperature on food consumption over temperatures from 20°C to 34°C, despite a three-fold decrease in average and maximum longevity between 20°C and 34°C. While there are likely a number of physiological constraints that may limit food intake at higher temperatures, these results were further corroborated by the lack of a relationship between individual longevity and food consumption in individual flies maintained at 25°C. A previous study of *C. stygia* also found individual sucrose consumption rates to be unrelated to their longevity [7], which strengthens the findings of the present study.

The examination of temperature effects on ageing and longevity may have been previously undervalued because of assumptions that temperature-induced responses in ectotherms were mainly a consequence of ‘rate of living’ effects [60]. This is unfortunate, as thermal perturbation studies of ectotherms provide a potentially powerful tool for examining the processes of ageing [17]. For example, we found that thermal history had a strong impact on the level of mortality that flies experienced when first transferred to a different temperature, however their rate of ageing after this transfer reflected the new temperature conditions. Flies transferred from high to low temperature showed a mid-life ‘suspension’ of senescence that was similar to the ‘negligible’ senescence of low temperature flies in the constant temperature experiment. The ability to induce this reduced senescence after previous ‘fast’ ageing, has the potential to present significant insights into the processes involved in ageing and longevity.

Mortality patterns of *D. melanogaster* under temperature or dietary restriction regimes were examined in a crossover experiment by Mair et al. [22]. The two environmental manipulations differed in their demographic ageing responses, while thermal history played an important role in determining mortality rates post-switch, under dietary restriction conditions, the current diet condition was the only factor that contributed to post-switch mortality. Their results reveal the influence of thermal history on longevity, but differ from our findings for *C. stygia* in an important way. In contrast to the slowed rates of ageing at low temperature observed in *D. melanogaster* [22], we found *C. stygia* to have similar rates of ageing at old age regardless of their temperature conditions.

The ‘suspension’ of senescence observed after transfer to low temperature likely reflects a temperature-sensitive mechanism that either decreases the rate of damage or increases the rate of repair, or perhaps a combination of both. To examine this, we measured the accumulation of fluorescent AGE pigment as a marker of cellular senescence and age-related damage over the life span of *C. stygia*. Increased rates of accumulation of various endogenous auto-fluorescent compounds with increasing temperature have been found in annual fish, *Nothobranchius spp.* [61], [62], milkweed bugs, *O. fasciatus* [33], *C. elegans* [39] and *Drosophila* [17], [28], [37]. Jacobson et al. [17] investigated six markers of oxidative damage in *D. melanogaster*, and found a strong correlation between the rate of mortality and fluorescent AGE pigment, but not in the other five markers measured. Similarly, in the current study AGE pigment accumulated with age, with faster rates of accumulation at high temperature. However the results are less clear following temperature transfer, possibly due to limited times of sampling. There was a large increase upon transfer to high temperature after 28 days, and a slight decrease after transfer to low temperature after 14 days, however the other two transfers showed no obvious temperature effect. These relatively small changes in AGE pigment did not reflect the dramatic changes seen in demographic senescence and demonstrates a partial decoupling between cellular senescence and demographic senescence in *C. stygia*.

## Supporting Information

Figure S1 **Gender differences in longevity and mortality rates are more dramatic at low temperatures. S1 Fig. A and B. Average (A) and maximum (B) longevity of male and female** ***C. stygia*** **maintained at different temperatures over the range of 12°C to 34°C.** *** *P*<0.001 as determined by a t-test between genders for each temperature. S1 Fig. C-H. Mortality rates for each gender are plotted separately for each temperature treatment. Data are the average of a 5-day period (error bars are omitted for clarity). Lines represent the Gompertz model best-fit to that gender-specific data (as determined by an AIC comparison).
(DOCX)

Figure S2 **Fluorescent AGE pigment accumulation in separated body segments of** ***Calliphora stygia*** **maintained at different ambient temperatures. Graphs are of fluorescent AGE pigment accumulation in: head (A), thorax (B) and abdomen (C) of** ***C. stygia*** **. All values are means ± SEM (** ***N =*** **6).**
(DOCX)

Figure S3 **Fluorescent AGE pigment accumulation in** ***Calliphora stygia*** **collected as ‘alive’, ‘dying’ or ‘dead’. Flies were maintained at 25°C and collected at days 16 and 35.** At day 16, ‘dead’ blowflies had a significantly higher level of AGE pigment than blowflies collected ‘alive’, but was not statistically different to those collected ‘dying’. Both the ‘alive’ and ‘dying’ blowflies had an increase in AGE pigment by day 35 (P<0.01 for both), yet there was no increase in the ‘dead’ blowflies (P>0.05). As a result of this, there was no statistically significant difference between the groups measured at day 35. ‘Dying’ blowflies were determined as blowflies found on their backs and could not right themselves. ‘Dead’ blowflies were found dead within the cage and could have potentially died at any point within the previous 24 hours. ‘Alive’ flies were collected as those flying around the cage and highly active. Values are means ± SEM (*N* = 8 for ‘dead’ and ‘alive’ blowflies measured at 16 and 35 days, *N* = 6 for ‘dying’ blowflies measured at both 16 and 35 days). * represents a significant difference at P<0.05, ‘ns’ represents no significant difference.
(DOCX)
