# Supplementary material for: Effect of Temperature on the Rate of Ageing: An Experimental Study of the Blowfly Calliphora stygia
Source: PLoS One. 2013 Sep 3;8(9):e73781. doi: 10.1371/journal.pone.0073781 (PMC3760806; doi:10.1371/journal.pone.0073781)
Supplement: Figure S1 — Gender differences in longevity and mortality rates are more dramatic at low temperatures. S1 Fig. A and B. Average (A) and maximum (B) longevity of male and female C. stygia maintained at different temperatures over the range of 12°C to 34°C. *** P<0.001 as determined by a t-test between genders for each temperature. S1 Fig. C-H. Mortality rates for each gender are plotted separately for each temperature treatment. Data are the average of a 5-day period (error bars are omitted for clarity). Lines represent the Gompertz model best-fit to that gender-specific data (as determined by an AIC comparison). (DOCX) [file pone.0073781.s001.docx]

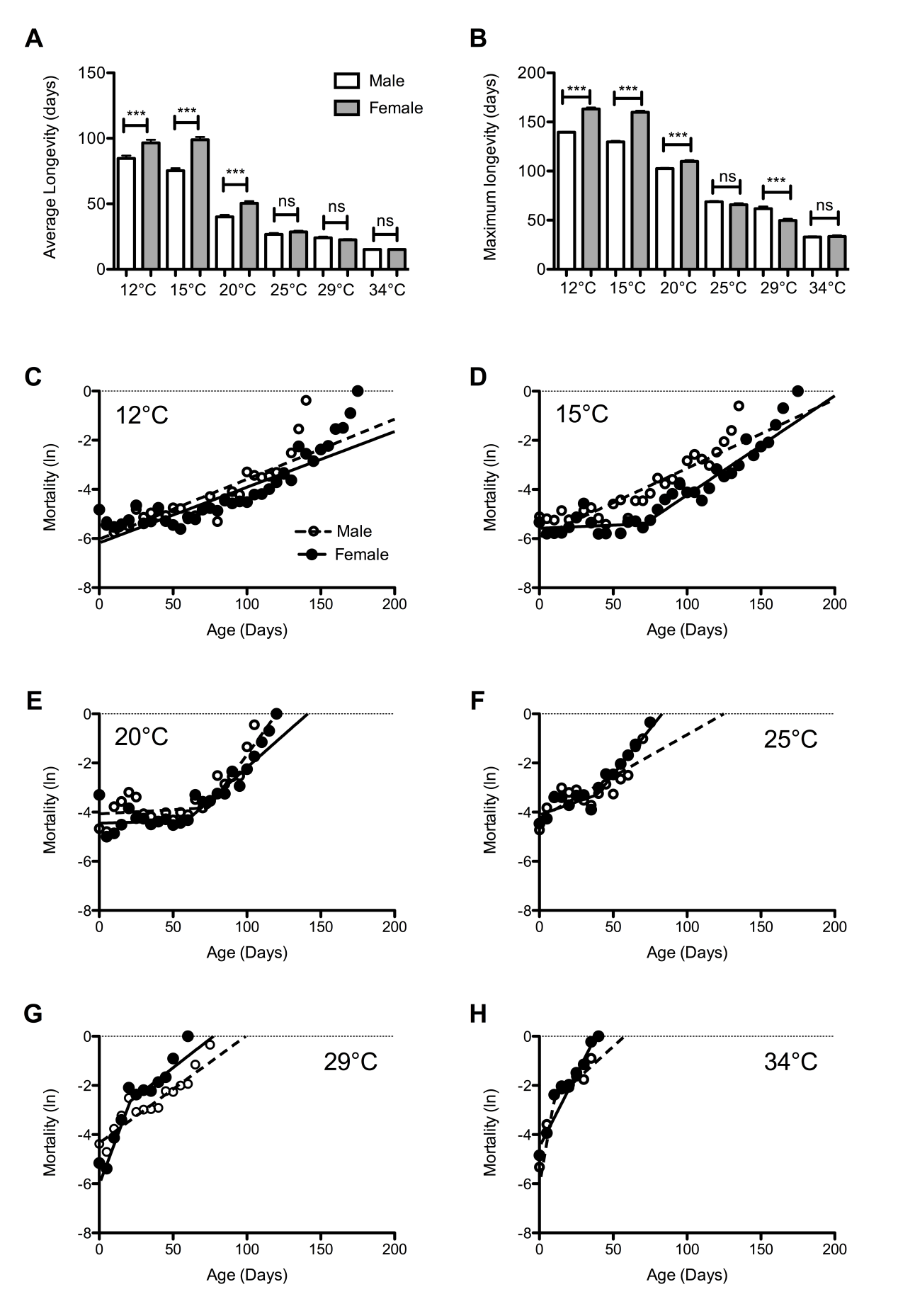


**Supplementary Figure S1. Gender differences in longevity and mortality rates are more dramatic at low temperatures. S1 Fig. A and B.** Average (A) and maximum (B) longevity of male and female *C. stygia* maintained at different temperatures over the range of 12°C to 34°C. *** *P* < 0.001 as determined by a t-test between genders for each temperature. S1 Fig. C-H. Mortality rates for each gender are plotted separately for each temperature treatment. Data are the average of a 5-day period (error bars are omitted for clarity). Lines represent the Gompertz model best-fit to that gender-specific data (as determined by an AIC comparison).
