# Supplementary material for: Effect of Temperature on the Rate of Ageing: An Experimental Study of the Blowfly Calliphora stygia
Source: PLoS One. 2013 Sep 3;8(9):e73781. doi: 10.1371/journal.pone.0073781 (PMC3760806; doi:10.1371/journal.pone.0073781)
Supplement: Figure S2 — Fluorescent AGE pigment accumulation in separated body segments of Calliphora stygia maintained at different ambient temperatures. Graphs are of fluorescent AGE pigment accumulation in: head (A), thorax (B) and abdomen (C) of C. stygia . All values are means ± SEM ( N = 6). (DOCX) [file pone.0073781.s002.docx]

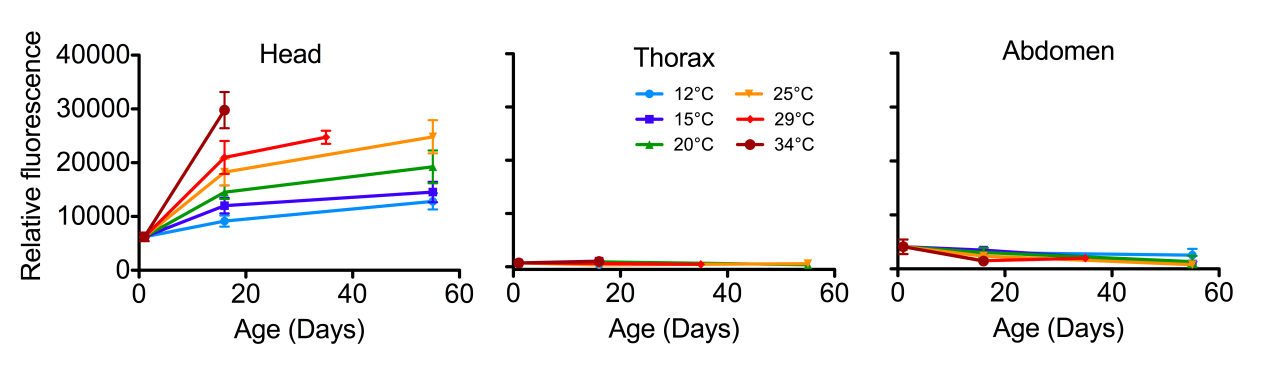


**Supplementary Figure S2**. **Fluorescent AGE pigment accumulation in separated body segments** **of *Calliphora stygia* maintained at different ambient temperatures**. Graphs are of fluorescent AGE pigment accumulation in: head (A), thorax (B) and abdomen (C) of *C. stygia*. All values are means ± SEM (*N* = 6).
