# Supplementary material for: Effect of Temperature on the Rate of Ageing: An Experimental Study of the Blowfly Calliphora stygia
Source: PLoS One. 2013 Sep 3;8(9):e73781. doi: 10.1371/journal.pone.0073781 (PMC3760806; doi:10.1371/journal.pone.0073781)
Supplement: Figure S3 — Fluorescent AGE pigment accumulation in Calliphora stygia collected as ‘alive’, ‘dying’ or ‘dead’. Flies were maintained at 25°C and collected at days 16 and 35. At day 16, ‘dead’ blowflies had a significantly higher level of AGE pigment than blowflies collected ‘alive’, but was not statistically different to those collected ‘dying’. Both the ‘alive’ and ‘dying’ blowflies had an increase in AGE pigment by day 35 (P<0.01 for both), yet there was no increase in the ‘dead’ blowflies (P>0.05). As a result of this, there was no statistically significant difference between the groups measured at day 35. ‘Dying’ blowflies were determined as blowflies found on their backs and could not right themselves. ‘Dead’ blowflies were found dead within the cage and could have potentially died at any point within the previous 24 hours. ‘Alive’ flies were collected as those flying around the cage and highly active. Values are means ± SEM (N = 8 for ‘dead’ and ‘alive’ blowflies measured at 16 and 35 days, N = 6 for ‘dying’ blowflies measured at both 16 and 35 days). * represents a significant difference at P<0.05, ‘ns’ represents no significant difference. (DOCX) [file pone.0073781.s003.docx]

**
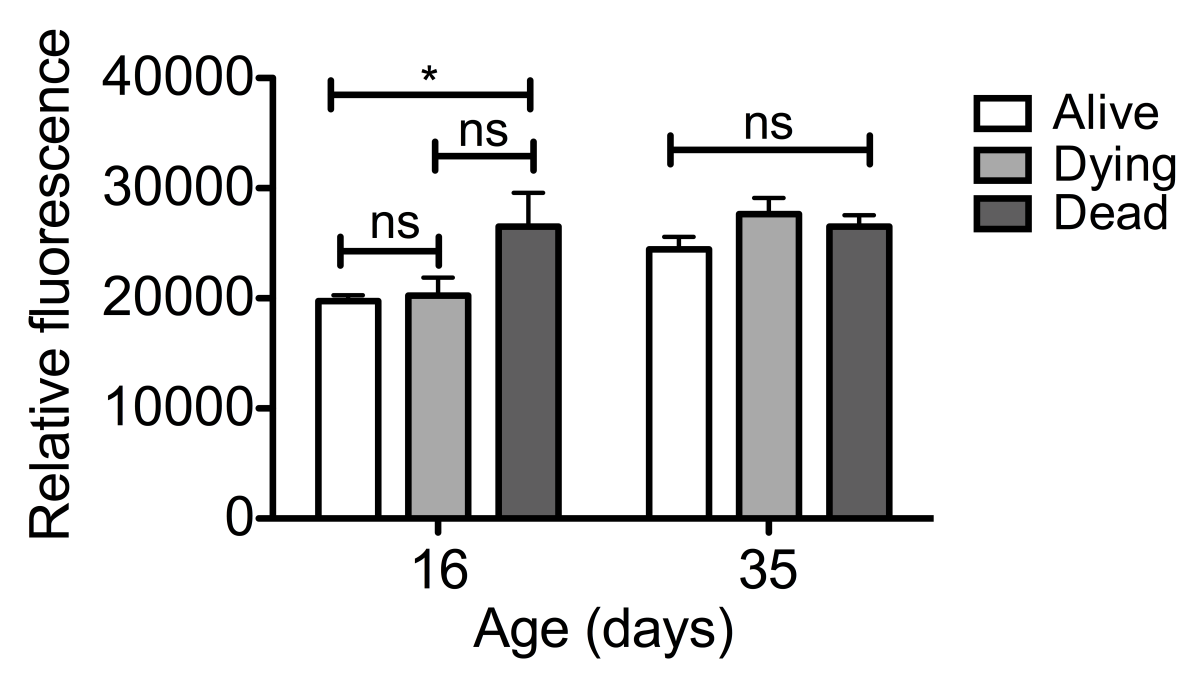
**

**Supplementary Figure S3. Fluorescent AGE pigment accumulation in *Calliphora stygia* collected as ‘alive’, ‘dying’ or ‘dead’**. Flies were maintained at 25°C and collected at days 16 and 35. At day 16, ‘dead’ blowflies had a significantly higher level of AGE pigment than blowflies collected ‘alive’, but was not statistically different to those collected ‘dying’. Both the ‘alive’ and ‘dying’ blowflies had an increase in AGE pigment by day 35 (P < 0.01 for both), yet there was no increase in the ‘dead’ blowflies (P > 0.05). As a result of this, there was no statistically significant difference between the groups measured at day 35. ‘Dying’ blowflies were determined as blowflies found on their backs and could not right themselves. ‘Dead’ blowflies were found dead within the cage and could have potentially died at any point within the previous 24 hours. ‘Alive’ flies were collected as those flying around the cage and highly active. Values are means ± SEM (*N* = 8 for ‘dead’ and ‘alive’ blowflies measured at 16 and 35 days, *N* = 6 for ‘dying’ blowflies measured at both 16 and 35 days). * represents a significant difference at P <0.05, ‘ns’ represents no significant difference.
